# Revisiting the Oxidation of Flavonoids: Loss, Conservation or Enhancement of Their Antioxidant Properties

**DOI:** 10.3390/antiox11010133

**Published:** 2022-01-07

**Authors:** Hernan Speisky, Fereidoon Shahidi, Adriano Costa de Camargo, Jocelyn Fuentes

**Affiliations:** 1 Laboratory of Antioxidants, Nutrition and Food Technology Institute, University of Chile, Santiago 7810000, Chile; adrianodecamargo@inta.uchile.cl; 2 Department of Biochemistry, Memorial University of Newfoundland, St. John’s, NL A1B 3X9, Canada; fshahidi@mun.ca; 3 Faculty of Medicine, School of Kinesiology, Universidad Finis Terrae, Santiago 7501015, Chile

**Keywords:** antioxidants, flavonoid oxidation, benzofuranones, flavonoids

## Abstract

Flavonoids display a broad range of health-promoting bioactivities. Among these, their capacity to act as antioxidants has remained most prominent. The canonical reactive oxygen species (ROS)-scavenging mode of the antioxidant action of flavonoids relies on the high susceptibility of their phenolic moieties to undergo oxidation. As a consequence, upon reaction with ROS, the antioxidant capacity of flavonoids is severely compromised. Other phenol-compromising reactions, such as those involved in the biotransformation of flavonoids, can also markedly affect their antioxidant properties. In recent years, however, increasing evidence has indicated that, at least for some flavonoids, the oxidation of such residues can in fact markedly enhance their original antioxidant properties. In such apparent paradoxical cases, the antioxidant activity arises from the pro-oxidant and/or electrophilic character of some of their oxidation-derived metabolites and is exerted by activating the Nrf2–Keap1 pathway, which upregulates the cell’s endogenous antioxidant capacity, and/or, by preventing the activation of the pro-oxidant and pro-inflammatory NF-κB pathway. This review focuses on the effects that the oxidative and/or non-oxidative modification of the phenolic groups of flavonoids may have on the ability of the resulting metabolites to promote direct and/or indirect antioxidant actions. Considering the case of a metabolite resulting from the oxidation of quercetin, we offer a comprehensive description of the evidence that increasingly supports the concept that, in the case of certain flavonoids, the oxidation of phenolics emerges as a mechanism that markedly amplifies their original antioxidant properties. An overlooked topic of great phytomedicine potential is thus unraveled.

## 1. Introduction

Controlling the rates of formation and removal of reactive oxygen species (ROS) is a dually essential function. On one hand, it is needed to secure the intracellular levels of ROS required to perform various biological functions, and on the other hand, to prevent exceeding such levels from reaching cytotoxic concentrations [1,2,3,4,5]. When the latter control goal fails, an oxidative stress condition ensues that, if stringent and sustained, will ultimately trigger a number of disease-leading molecular events [6,7].

To maintain ROS below deleterious levels, cells are naturally endowed with a series of enzymes whose functions include the removal of ROS via either dismutation (e.g., superoxide dismutase, SOD; catalase, CAT), catabolic (e.g., heme oxygenase-1, HO-1) or reduction reactions (e.g., glutathione peroxidase, GSHpx; NAD(P)H:quinone oxidoreductase 1, NQO1), synthesizing endogenous ROS-scavenging/reducing molecules (e.g., reduced glutathione via gamma glutamate-cysteine ligase, Ɣ-Glu–Cys ligase), or regenerating cofactors needed by some ROS-reducing enzymes (e.g., reduced glutathione, GSH, via glutathione reductase, GSSGred).

In addition to this cooperative array of enzyme-based antioxidant defense mechanisms, cells contain a number of non-enzymatically acting antioxidant molecules, of which reduced glutathione (GSH), ubiquinol, dehydrolipoic acid, melatonin, ferritin, caeruloplasmin, and metallothioneins are endogenously synthesized [8], while α-tocopherol, ascorbic acid, carotenoids and phenolics are acquired through dietary sources [9]. Among the latter molecules, academia and industry have paid a great deal of attention to phenolics, particularly flavonoids, due to their comparatively higher antioxidant capacity and ubiquitous presence in edible plants [10,11].

## 2. Flavonoids as Antioxidants

Flavonoids have attracted the attention of biomedical researchers due to their potential to induce an array of health-promoting biological actions [12,13,14,15]. Major support for the potential health benefits of these compounds initially emerged from epidemiologic studies conducted in the 1990s. At that point, inverse correlations between the intake of flavonoid-rich foods and the relative risk of developing certain chronic noncommunicable diseases (NCDs) were established [16,17,18,19,20,21]. Over the last two decades, however, the conclusions arising from those population-based studies have gained support through a number of animal studies, in vitro cell mechanistic investigations and human intervention studies [19,22,23,24,25,26,27]. Comprehensive reviews on the health effects of dietary flavonoids have appeared in recent years [15,28,29,30,31].

**Figure 1:**
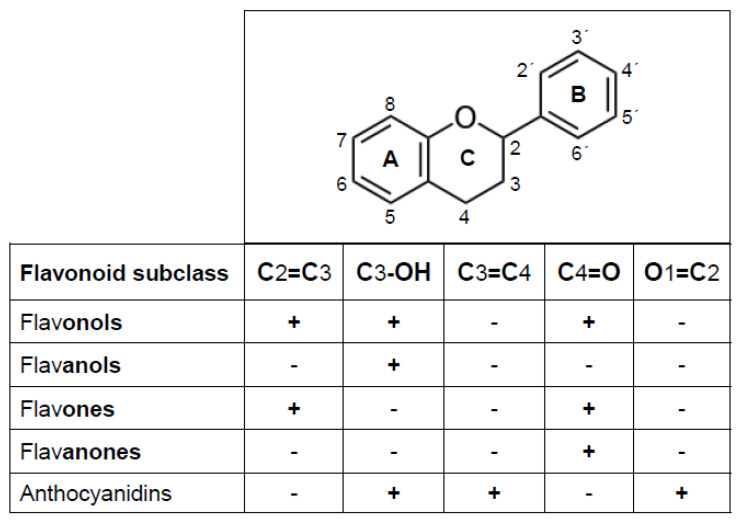
Flavan nucleus, 2-phenyl-3,4-dihydro-2H-1-benzopyran skeleton, common to all flavonoids (C6–C3–C6).

Near eight thousand flavonoids have been described to date in the plant kingdom [11]. The systematic study of those of dietary origin has led to the development of several reports and/or databases that inform on their contents in foods and dietary level of consumption, and their biotransformation and bioavailability [32,33,34,35]. From a chemical point of view, the term flavonoid comprises all those molecules whose structural backbone (a flavan nucleus, C6–C3–C6, Figure 1) consists of two benzene rings (A and B) that are linked through three carbon atoms that form a pyran heterocyclic ring (C). This structure allows multiple patterns and substitutions that give rise to various subclasses of flavonoids, among which flavonols, flavones, flavanones, flavanols and anthocyanidins can be distinguished. Such categorization is based on whether the flavan nucleus contains a hydroxyl moiety in C3 (i.e., flavonols, flavanols and anthocyanidins), a keto group in C4 (i.e., flavonol, flavones and flavanones), a double bond in C2–C3 (i.e., flavonols and flavones), a double bond in O1–C2 and another in C3–C4 (anthocyanidins).

In addition to flavonoids, there are isoflavonoids, mainly represented by the isoflavones, whose structure contains a double bond at C2–C3 and a keto group at C4. Isoflavones differ from flavonoids in that ring B is attached to C3 instead of C2. Regardless of the subclass, when the structure of a flavonoid includes one or more hydroxyl groups attached to its rings A and/or B, it is considered a phenolic compound [36]. Common hydroxylation points are at positions 5, 7 (A ring), 3′, 4′, 5′ (B ring), and 3 (C ring). Added to the structural features that define a flavonoid subclass, the number and position of the hydroxyl groups constitute a major determinant of the physicochemical characteristics and the myriad of biological actions displayed by these compounds [37,38]. In fact, depending on their structural particularities, flavonoids can display antioxidant, anti-inflammatory, anti-allergic, anti-platelet aggregation, anti-atherogenic, anti-angiogenic, anti-allergic, blood vessel-dilating, lipid-normalizing, antimicrobial and/or anti-hyperglycemic actions [26,39,40,41]. Among all bioactivities, the ability of flavonoids to act as antioxidants, namely as molecules capable of essentially lowering the rate of ROS formation and/or increasing the rate of their removal, is the only one shared by all flavonoids [42,43].

The ability of flavonoids to act in vitro as antioxidants, which primarily arises from the phenolic hydroxyls that are attached to the flavonoids’ flavan nucleus, has long been documented [38,44,45]. Comparatively, lesser but still substantial evidence also exists for the ability of these compounds to exert some antioxidant actions in vivo. In fact, a number of studies in humans and animals have revealed that the increase in several markers of biological oxidation induced by ROS, such as F2-isoprostanes, hydroperoxyoctadecadienoic acids, 8-hydroxy-2′-deoxyguanosin, oxidized low density lipoprotein, nitrotyrosine and other nitrosylated or carbonylated amino acids and proteins, can be effectively prevented or ameliorated by the ingestion of certain flavonoid-rich plant foods or the administration of either flavonoid-rich extracts or pure flavonoids, as reviewed by several authors [46,47,48,49]. The broad recognition of the latter effects of flavonoids is likely to account for the so generalized and long perception that “flavonoids act primarily as antioxidant molecules”.

The contribution of flavonoids to the cell’s antioxidant capacity can potentially be exerted through a number of distinctive mechanisms, as reviewed by several authors [42,50,51,52]. In general, however, most studies have drawn their attention to the ability of flavonoids to interact via their redox-active phenolic moieties with a variety of ROS and/or target molecules that are implicated in the formation and/or removal of these species. Regardless of the antioxidant action mechanism of flavonoids, one of the ultimate consequences that such action will bring to the cells is to prevent oxidative stress or left the cells metabolically better able to deal with it.

In addition to the changes in the antioxidant capacity of the cell induced by flavonoids and depending on the mechanism involved, the flavonoid molecule could itself undergo no changes in its structure or be chemically modified in a manner that could severely affect its original antioxidant properties. An example of the latter case would be illustrated by the loss of antioxidant activity suffered by those flavonoids whose actions are exerted by scavenging/reducing ROS, an operative mechanism that fully depends on the integrity of the redox-active phenolic moieties present on the flavonoid’s structure [53]. It has been generally believed that the oxidative consumption of the phenolic moieties implied in the ROS scavenging/reducing mode of action would necessarily compromise or lead to the loss of such antioxidant properties of the flavonoid. However, during the last two decades, considerable evidence has emerged, indicating that, at least for certain flavonoids, the oxidation of their phenolic moieties would be essential for them to subsequently exert an antioxidant action [54,55,56]. Thus, rather than the flavonoid molecule, one (or more) of its metabolites arising from its oxidation would serve as the actual active antioxidant species.

As we have recently shown [53], the mixtures of metabolites originating from the oxidation of certain flavonoids largely retained rather than lost the ROS scavenging/reducing properties of their parent molecules. Furthermore, it has been unveiled that in some particular cases, the flavonoid oxidation mixture contains a type of metabolite that is able to protect cells against ROS or ROS-inducing agents, with a potency two-to-three orders of magnitude higher than that of its precursor flavonoid [57]. This latter evidences the existence of two apparently contrasting views, one that highlights the need for flavonoids to occur in their non-oxidized form to be effective as ROS-scavengers and another where their prior oxidation appears to be fundamental to the retention or even amplification of their antioxidant action. To address the question of whether the oxidation of a flavonoid leads to loss, the conservation or enhancement of its antioxidant properties, in this review, we mostly focused our discussion on studies where, at least for some of these compounds, the oxidation of (or other forms of compromising) their redox-active phenolic moieties, rather than eliminating their original antioxidant properties, can operate as a major antioxidant-activating mechanism.

## 3. Oxidation and Other Metabolic Reactions Capable of Affecting the Antioxidant Properties of Flavonoids

The best characterized mechanism of antioxidant action of flavonoids is due to their ability to interact with ROS by scavenging or reducing them. In this canonical direct mechanism, the redox-active phenolic moieties of a flavonoid molecule engage with ROS to a redox reaction where as a consequence of its scavenging action, an electron or a hydrogen atom is transferred from such moieties [58,59]. Based on a generally large body of in vitro evidence, for a long time—between the 1980s and early 2000s—the ROS scavenging/reducing action of flavonoids was assumed to be the main mechanism by which these compounds exerted their antioxidant actions in vivo [60,61,62]. More recently, however, such an assumption has been increasingly questioned [42,63,64,65,66], including kinetic and thermodynamic considerations [42,67,68]. However, a major argument against the possibility that the ROS-scavenging/reducing mechanism could account for their in vivo antioxidant effects of flavonoids arose after establishing a near two orders of magnitude difference between the concentrations of many flavonoids needed to act as ROS-scavengers/reducing in vitro (low micromolar) and those actually attained in plasma (low-to-medium nanomolar) after the ingestion of foods rich in such flavonoids [69,70,71]. It should be noted, however, that a direct ROS-scavenging action of flavonoids could be more relevant in those anatomical sites that are more directly exposed to them, such as the mucosa of the gastrointestinal (GI) tract, and eventually, the skin after their deliberate direct application to this tissue.

A second mechanism of the antioxidant action of flavonoids, in which the oxidation of its phenolic moieties is also involved, is an “indirect mechanism” where these compounds do not directly interact with ROS but with certain proteins that, via the regulation of gene expression, ultimately upregulate the cell’s endogenous antioxidant capacity [55,67]. In this mechanism, the oxidation of some of the flavonoid’s phenolic moieties would constitute a step needed to subsequently exert its antioxidant action. Thus, the antioxidant action is not triggered by the flavonoid molecule itself but through a metabolite that results from its oxidation [54,55,56,72]. However, it should be noted that for those flavonoids that act as antioxidants in vitro through a gene expression-regulating mechanism, the needed concentrations are also within a low-to-medium micromolar range. Since, in this indirect mechanism, an oxidized metabolite exerts the antioxidant action, its concentration in plasma or in the target tissues, and not that of the flavonoid, would be the one to be taken into consideration. Unfortunately, to the best of our knowledge, neither in vivo nor in vitro studies have addressed such a fundamental issue to date.

There is a consensus that the nanomolar concentrations of flavonoids found in the systemic circulation reflect the low oral bioavailability of these compounds and that, in general, this latter is attributable to their poor GI absorption and, overall, to their extensive biotransformation [73,74,75,76]. Prompted by the large in vitro versus in vivo flavonoid concentration gap, several investigators have pointed out that rather than the flavonoids themselves, some metabolites that are generated during their biotransformation and/or oxidation could account for their in vivo antioxidant effects [66,72,77,78,79,80]. Within such a conceptual frame, one might reason that if the metabolites formed in vivo conserved the same antioxidant potency shown by their precursors in vitro, such metabolites would need to circulate in plasma at micromolar concentrations. Alternatively, if the metabolites circulate in plasma at concentrations comparable to those attained by their precursors, the former will need to exhibit an at least two orders of magnitude higher ROS-scavenging or antioxidant gene expression-regulating potency.

Several biochemical processes that are involved in the metabolic handling of flavonoids end up affecting their chemical structures, physicochemical properties and, potentially, their bioactivities, including the antioxidant effect (Table 1). In general, flavonoids occur in edible plants largely in their *O*-glycosylated form, bound to sugar moieties such as glucose, rhamnose or galactose. The *O*-glycosides of flavonoids are found in edible plants, mainly as 3 or 7 *O*-glycosides, although the 5, 8 and 4′ *O*-glycosides have also been reported in some cases [81]. One of the earliest processes that affect the structure of flavonoids after their ingestion is their deglycosilation during the transit along the gastrointestinal tract. This step is critical in the absorption and metabolism of dietary flavonoid glycosides in human subjects [82]. Whether ingested as a food component or a pure glycoside, these compounds are hydrolyzed to aglycones by glycosidases present in the brush border membranes (i.e., lactase-phlorizin hydrolase) or the cytosol (i.e., β-glucosidase) of the small intestine epithelial cells, and principally, in colon-residing microbiota [83,84]. Subsequently, most flavonoid aglycones are subject to biotransformation, a process that, through phase I (e.g., oxidation, demethylation) and preferentially phase II (e.g., methyl-, sulpho- and glucuronyl-conjugation) reactions, significantly modifies their structures and potentially their antioxidant properties. This process can take place pre-systemically, during the diffusion of the flavonoids through the epithelial cells of the proximal small intestine, during their subsequent first-pass through the liver, and/or after reaching the colon through the action of biotransforming enzymes present in the microbiota. Upon entering the circulation, the flavonoid aglycones and/or their phase I/II metabolites can undergo further biotransformation systemically, during all the post-absorption phases, namely distribution, metabolism and excretion [22,85,86,87,88,89]. In the case of some flavonoids (anthocyanidins are an exception), the effect of the pre-systemic phase II biotransformation can be so significant that, following their intestinal absorption and transport to the liver via the portal vein, they circulate in systemic blood almost exclusively as *O*-glucuronide, *O*-sulphate and/or *O*-methyl ester/ether metabolites (generally in this order of abundance) [69,90].

In addition to its bioavailability-lowering effect, the biotransformation process often enhances the polarity of its substrates, accelerating their elimination. An apparent exception for the latter is the one that affects flavonoids such as quercetin whose conjugation metabolites, after reaching (or being formed in) the liver, are biliary excreted back into the duodenum from where they undergo enterohepatic recirculation (e.g., quercetin glucuronides) [91,92]. However, even in such a case, it has been established that after the ingestion of a large portion of quercetin-rich vegetables, the peak plasma concentrations of its individual conjugates only fall within the low-to-medium nanomolar range [93,94,95]. Although phase II conjugation reactions take place along the intestinal absorption of flavonoids affect, in general, the bioavailability of their aglycones, some studies have pointed out that, at least for quercetin, its 3-glucuronide could undergo deconjugation in vascular tissues with inflammatory injuries [96]. It has been shown that this metabolite accumulates in atherosclerotic lesions and within macrophage-like foam cells, from where it is deconjugated by β-glucuronidase, leading to a biological effect of endothelium function [97]. Hence, quercetin-3-glucuronide has been proposed to behave as a quercetin carrier in plasma, which deconjugates in situ, releasing the aglycone. However, the occurrence of deconjugation in vessels for other flavonoids remains to be investigated.

Regarding the effects of biotransformation on the antioxidant activity of flavonoids, although neither the exact direction nor the magnitude of a change in such activity can be precisely predicted on the sole basis of the chemical nature of a flavonoid [98], theoretically, it can be expected that nu blocking via methylation, sulfation or glucuronidation, one or more of its redox-active phenolic groups, for instance, a single phenolic, catechol or galloyl in ring B, would compromise the flavonoid’s original antioxidant properties [61,99,100]. In fact, most studies indicate that when such a type of metabolites are assayed in vitro for their ROS-scavenging/reducing activity, these have either significantly lost or only marginally retained the antioxidant activity of their precursors, but that in no case have they undergone a substantial gain of such activity [74,96,101,102,103,104,105,106,107,108,109,110,111,112]. Essentially, similar in vitro results have recently been reported regarding the capacity of some flavonoids’ phase II-conjugation metabolites to upregulate (through an indirect action) the cell’s endogenous antioxidant capacity [80,113,114,115] (Table 1). It should be noted, however, that in some particular cases, phase I and/or II biotransformation metabolites have been shown to exert a number of other, not necessarily antioxidant-dependent, biological actions that could significantly contribute to the health-promoting effects of their precursor flavonoids [79,116,117].

A second process that can substantially compromise the structure of flavonoids, and thereby influence the plasma circulating concentration and/or the antioxidant properties of the generated metabolites, is the one that affects the fraction of the ingested flavonoids that during their gastrointestinal transit was not intestinally absorbed, and that, upon reaching the colon, undergoes substantial microbiota-mediated catabolism [84,118,119,120,121]. In fact, in recent years, important advances have been made in defining the catabolic capacity and structure-modifying effects of the gut microbiota on distinct flavonoids, and in parallel, how flavonoids can affect the composition and biological activity of such bacteria [121,122]. The enzymes present in the colonic microbiota catalyze not only the degradation of some flavonoid aglycones through C-ring cleavage, demethylation and/or dehydroxylation reactions, but also that of many flavonoid glycosides, through *O*-deglycosylation and ester hydrolysis, and that of phase-II conjugates, through the action of β-glycosidases [123]. The former processes can convert flavonoids into a broad set of lower molecular weight catabolites [124], of which most are simpler phenolics and aromatic acids that appear in the blood and circulate in their free state or as (colon-generated) phase II conjugated catabolites. Several researchers have proposed that the bioactivities of some of these catabolites, which are not necessarily associated with antioxidant actions, could account for at least part of the beneficial health effects attributed to their precursors [41,119,122,125]. Interestingly, it has been reported that some colonic catabolites can reach high micromolar concentrations in fecal water [126,127], from where they could be readily absorbed to reach, in some specific cases (i.e., catechol- and pyrogallol-sulphates), low micromolar concentrations in systemic circulation, namely, concentrations that are thus notably higher than those attained by their parent flavonoids and/or by their corresponding flavonoid conjugates [128]. Owing to the latter, it has been proposed that, at least part of the antioxidant effects of flavonoids seen in vivo might be ascribed to some of their systemically circulating colonic catabolites [121,129,130]. However, most in vitro studies indicated that the ROS-scavenging/reducing potency of such catabolites is only either slightly [100] or substantially lower [131] than that of their precursors. A possible exception of the latter would be that of some colonic catabolites whose structure conserves the catechol moiety of their precursor flavonoids, as has been suggested to either retain or exhibit an even slightly higher ROS-scavenging/reducing activity compared to their precursors [124,129]. On the other hand, although some colonic catabolites derived from flavonoids have been reported to also be able to upregulate the activity of several ROS-controlling enzymes [132,133], the in vitro concentrations needed to elicit such effects ranging from 25 to 250 micromolar, which are reportedly unlikely to be found in plasma after the ingestion of flavonoids.

The third type of process that compromises the structure of flavonoids, and that could potentially lead to a change in their antioxidant properties, refers to the oxidation that their phenolic groups undergo during their interaction with ROS, with certain oxidizing enzymes, or with other molecules whose structures contain chemical residues that are susceptible to be reduced by the redox-active phenolic moieties of flavonoids. Considering the scope of this contribution, this specific structural modification will be addressed in the following section.

## 4. Oxidation of the Phenolic Moieties of Flavonoids and Its Consequences on Their Antioxidant Properties

As already mentioned, the oxidizability of the phenolic moieties of all flavonoids is the basis for their ability to either scavenge or reduce different ROS. During such reactions, one (or more) of the phenolic groups engages in a redox reaction where either an electron or a hydrogen atom of a hydroxyl groups is transferred to the ROS, stabilizing these species [58,59]. The latter reaction, as described in more detail below for quercetin, necessarily converts the flavonoid into a free radical intermediate, ultimately giving place to the formation of an oxidized metabolite, or to a set of different metabolites. In this mechanism, the ROS-scavenging action of the flavonoid would last as much time as it takes to oxidatively consume its redox-active phenolic groups. However, it remains to be seen if, after undergoing such oxidation, the flavonoids that act through this direct antioxidant mechanism will necessarily lose their original antioxidant properties. The answer to this question was, for a long time, positive. The reason for that was that in order to function as a directly acting antioxidant, the redox-active phenolic groups of a flavonoid involved in its ROS scavenging/reducing action need to exist in their reduced state. Consequently, if such groups have already engaged in a reaction where they have been oxidatively consumed, it seems reasonable to assume that the generated metabolite(s) will necessarily be devoid of the flavonoid’s original ROS scavenging/reducing ability. Similarly, this argument might be extended to those flavonoids whose original structures need to be preserved in order to inhibit the catalytic activity of ROS-generating enzymes and/or to chelating redox-active metals. Recently, however, some evidence has emerged revealing that such contention needs to be revised—at least for the ROS-scavenging and ROS-reducing capacity of certain flavonoids. In fact, in addressing the consequences that the oxidation of quercetin and that of thirteen other structurally related flavonoids could bring on, in terms of their original ROS-scavenging (ORAC assay) and ROS-reducing (Folin–Ciocalteu- and Fe-Triazine) properties, Atala et al. [53] reported that most of the mixtures of metabolites that resulted from such oxidations partially or largely conserved, rather than lost, the antioxidant properties of their precursors. These latter effects were seen regardless of the method employed to induce their oxidative consumption (i.e., alkali-induced or mushroom tyrosinase-mediated) and in the case of the alkali-exposed flavonoids, the oxidation mixtures of 9 of the 14 tested flavonoids (which included flavanols, flavonols, flavones and flavanones) exhibited ROS-scavenging remnant activities that were greater than 70%, and that thirteen of the 14 tested flavonoids retained over 50% of the original Folin–Ciocalteu-reducing properties. While the referred to study did not establish the chemical identity of the metabolites in each oxidation mixture, the authors speculated that the oxidation process would not grossly alter those structural moieties that are primarily responsible for the ROS-scavenging and/or redox-reducing properties of the flavonoids. Presumably, such moieties would comprise phenolic groups that are capable of stabilizing ROS and/or reducing the Folin–Ciocalteu reagent. However, other structural features that could be favorable in terms of stabilizing the resulting phenoxyl radical(s) are also likely to be present in the structure of the putative oxidation metabolites (i.e., electron-delocalizing and resonance-permitting moieties). Under the time-controlled alkali-induced oxidation conditions employed by Atala et al. [53], ten flavonoids (namely quercetin, myricetin, fisetin, dideoxyquercetin, taxifolin, eriodictyol, isorhamnetin, epicatechin, luteolin and catechin) had almost completely disappeared. Out of these, the four flavonoids that almost completely retained their original ROS-scavenging activity were the flavonols quercetin, dideoxyquercetin, isorhamnetin and fisetin, whose structures simultaneously include either one or two unsubstituted hydroxyl groups in ring B, and an enol moiety (i.e., C2–C3 double bond with a C3-hydroxyl) in ring C. In turn, flavonoids that have a catechol in ring B but lack a double bond in the C2–C3 position of ring C (flavanols and flavanones) exhibited the lowest degree of antioxidant retention (i.e., catechin, epicatechin, eriodictyol, and taxifolin). In addition to its antioxidant-retaining implications, the ability of the mixtures of oxidized flavonoids to scavenge ROS and/or reduce the Folin–Ciocalteu and Fe-triazine reagents might have some methodological implications [134]. That is, when a flavonoid is assayed using any of the previously mentioned (flavonoid-oxidizing) methods, a mixture of compounds is likely to be formed that could inadvertently contribute to the observed results. During the initial phase of oxidation, this mixture may comprise the reduced flavonoid plus several redox-active metabolites generated during the assay of the flavonoid, which could be particularly important when the sum of the ROS scavenging/reducing activities of such metabolites is comparable to that of the flavonoid from which they originate. In such cases, the antioxidant activity believed to strictly arise from the reduced flavonoid is likely to be overestimated, eventually limiting the interpretation of some structure–antioxidant activity relationship studies. However, prior to questioning the interpretation of such a study type, it should be considered that the composition as well as the degree of antioxidant capacity retained by any mixture of metabolites will depend, not only on the structural particularities of the flavonoid but also on the conditions employed to induce its oxidation and the method used to assay its antioxidant activity. Nonetheless, as discussed below, at least in the case of quercetin, it has been reported that, regardless of the experimental mode used to induce its oxidation, an essentially similar set of metabolites is always formed [135].

As already pointed out, during the last two decades, a growing body of evidence has emerged to reveal that, in addition to the ROS-scavenging/reducing mechanism of action, some flavonoids are also able to promote antioxidant effects via the previously mentioned indirect mechanism of action. In this mechanism, the flavonoid ultimately modulates the expression of certain genes that code for the synthesis of ROS-forming enzymes (inhibiting it) and/or ROS-removing enzymes (inducing it), and/or by upregulating the expression of genes that code for antioxidant-synthesizing enzymes. The most commonly reported mediator of these indirect antioxidant actions is the redox-sensitive transcription protein, nuclear factor (erythroid-derived 2)-like 2 (Nrf2), that regulates the expression of a large number of genes that contain an enhancer sequence in their promoter regulatory regions termed antioxidant response elements (AREs), or probably more accurately named, electrophile-response elements (EpRE) [67,136,137]. The regulation of the Nrf2 pathway is mainly mediated by the interaction between Nrf2 and its cytoplasmic repressor Kelch-like ECH-associated protein 1 (Keap1), an E3 ubiquitin ligase substrate adaptor that under physiological or unstressed conditions targets Nrf2 for rapid ubiquitination and proteasomal degradation, resulting in a limited cytoplasmatic concentration of Nrf2 [138,139]. Keap1 contains, however, several highly reactive cysteine residues that, upon undergoing conformational modification, facilitate the swift translocation of Nrf2 into the nucleus (i.e., Nrf2-Keap1 activation). Although some of the critical cysteines in Keap1 can be directly oxidized or covalently modified, the Nrf2–Keap1 pathway can also be modulated by the transcriptional modification of Nrf2, particularly via phosphorylation by a series of redox-sensitive protein kinases such as the extracellular signal-regulated protein kinase (ERK1/2), protein kinase C (PKC) and c-Jun N-terminal kinase (JNK) [140,141]. Following its translocation into the nucleus, Nrf2 undergoes dimerization with small musculoaponeurotic fibrosarcoma oncogene homologue (sMAF) proteins. The heterodimers thus formed induce the de novo synthesis of a variety of proteins that are encoded in the ARE/EpRE-containing genes. The activation of the Nrf2-dependent ARE/EpRE signaling pathway translates into increasing the cells’ enzymatic (e.g., SOD, CAT, GSHpx, NQO1, HO-1) and non-enzymatic (e.g., GSH) antioxidant capacity [142,143,144,145,146,147,148] and/or its capacity to conjugate a broad range of electrophiles via phase II biotransformation enzymes (e.g., glutathione S-transferases, UDP-glucuronosyltransferases) [149]. Although under normal conditions the Nrf2–Keap1 pathway plays an essential role in maintaining the intracellular redox homeostasis, substantial evidence indicates that its activation by certain ROS and/or by a large number of electrophiles is pivotal to protect cells from the detrimental effects associated with the intracellular accumulation of these species [150,151,152]. An early Nrf2 activation by low concentrations of certain ROS and/or electrophiles would protect cells not only by preventing them undergoing the otherwise redox-imbalance (oxidative stress) expected to arise from a sustained accumulation of ROS, but also by preventing the covalent binding of electrophiles to DNA and certain proteins whose normal functioning is vital to cells. Compared to the antioxidant effects that arise from the ROS-scavenging/reducing actions of flavonoids, those resulting from the activation of Nrf2 require a lag time to manifest but are comparatively longer lasting since their duration is essentially defined by the half-lives of de novo synthesized antioxidant enzymes. Additionally, due to the catalytic character of any enzyme, the antioxidant effects of flavonoids exerted via this indirect mechanism are amplified and manifested beyond the time-restricted action of the direct acting flavonoids whose antioxidant effects are limited by their stoichiometric oxidative consumption. Cumulative experimental evidence [153,154], and more recent evidence provided by several clinical trials [155,156], indicate that molecules that are able to induce the activation of Nrf2 could become an effective means to prevent and/or treat a number of pathological and/or toxicological conditions whose common etiological denominator is the early and sustained occurrence of oxidative stress [157,158].

Although Nrf2 activators comprise a large group of structurally distinct molecules, oxidizable diphenols have emerged among the earliest ones discovered [159]. Particular attention was initially placed on simple catechols (1,2-diphenols) and hydroquinones (1,4-diphenols) since these compounds are able to readily participate in one- or two-electron reversible oxidation reactions giving rise to electrophilic *ortho*- and *para*-quinones, respectively [160,161]. Due to their ability to avidly react with sulfhydryl groups, these phenol-derived electrophilic species are able to ultimately modify, via either oxidation, alkylation, or thiol-disulfide interchange reactions, some of the critical redox-sensitive cysteine residues in Keap1 [54,137,162]. Since the electron-deficient core of these quinones can also easily react with nucleophilic thiols present in other cysteine-containing proteins and/or with the sulfhydryl moiety of glutathione, such interactions can be potentially deleterious when the electrophiles occur within cells at high concentrations [163]. At low nanomolar intracellular concentrations, however, the formation of phenol-derived quinoids is only associated with an increase in the so-called ‘nucleophilic tone’ of the cells [42]. In addition to certain phenolic alcohols and acids, a great deal of attention has been placed in recent years on other compounds, among which terpenoids, isothiocyanates, indoles, organo-sulfides, curcuminoids, stilbenes, chalcones and flavonoids are included. In the case of flavonoids, the list of compounds capable of acting as Nrf2 activators comprises specific congeners of each of the six flavonoids subclasses [164,165,166]. Although flavonoids do not have electrophilic activity as such, in some cases, their oxidation leads to the formation of electrophilic and/or pro-oxidant metabolites [167]. Particularly, flavonoids that exhibit a 1,2- or a 1,4-diphenol, or a galloyl moiety (1,2,3-triphenol) in the B ring, but not the mono- or 1,3-diphenol variants, have a higher probability of being readily oxidized to semiquinones and quinones, resulting in redox cycling and production of ROS, of which both chemical species could potentially react with the sulfhydryl moiety of certain Keap1-contained cysteines [168,169]. Early work by Lee-Hilz et al. [54] showed that the ability of certain flavonoids to activate an ARE/EpRE-mediated antioxidant response correlates well with their redox properties characterized by quantum mechanical calculations, that flavonoids with a higher intrinsic potential to generate oxidative stress and/or redox cycling are the most potent inducers, and that activation exerted by flavonoids increases after decreasing the intracellular GSH and vice versa, supporting an oxidative mechanism. Recognition of all the latter is coherent with the contention that rather than the flavonoid itself, the ultimate Nrf2-activating species would be the flavonoids’ electrophilic metabolites, or alternatively, the ROS derived from the potential of its quinones to undergo redox cycling [42,54]. As shown by Zoete et al. [170], the HOMO energy or electron-releasing power (i.e., the easiness with which a molecule donates an electron and oxidizes) of 30 different polyphenols correlated well with their capacity to induce the EpRE-mediated gene transcription of NQO1, a phase II detoxifying enzyme known to be induced by Nrf2. In line with such results, Lee-Hilz et al. [54] also reported that the HOMO energy of 21 different flavonoids correlated well with their induction factor for the EpRE-mediated gene transcription. According to these latter investigators, flavonoids with a higher intrinsic potential to generate oxidative stress and redox cycling are the most potent inducers of EpRE-mediated gene expression. Over the last decade, a considerable number of studies has demonstrated the ability of some specific flavonoids to induce, via the activation of the Nrf2–Keap1 system, the expression of antioxidant and phase II detoxifying enzymes, in diverse cell models. Such an ability would reside in the capacity of such flavonoids to undergo enzymatic and/or non-enzymatic oxidation reactions that, at some point, convert them into electrophilic quinoid species (e.g., semi-quinones, and quinone methides) and/or certain ROS [171,172,173]. The latter species can be generated during the interaction of some specific flavonoids (i.e., diphenols) with: (i) certain ROS (e.g., superoxide anions, hydroxyl and peroxyl radicals) since after scavenging or reducing them, the flavonoids are converted into phenoxyl radicals and potentially into quinoid species; (ii) catalytic concentrations of some redox-active transition metals which in, their reduced state (e.g., Cu^1+^ or Fe^2+^) and, presence of oxygen will generate superoxide anions that subsequently, via dismutation, will form hydrogen peroxide; and (iii) certain metalloenzymes (e.g., peroxidases, tyrosinases, oxidoreductases) that are able to catalyze their oxidation, leading to the formation of semiquinones and quinones. In the case of quercetin, shown to accumulate in large amounts within mitochondria [174], the formation of its quinone/quinone methide metabolites has been reported to take place not only in peroxidase containing cell-free systems [175] but also in tyrosinase-rich cells (i.e., B16F-10, a mouse melanoma cell line) [171]. According to Awad et al. [171], the intracellular formation of these quinoid species could also take place in other mammalian cells known to contain peroxidase-like activities.

Flavonoids that carry two or more hydroxyl moieties in their B ring are recognized to be more prone to form quinoid intermediates, and consequently rank highest among the Nrf2-inducers. It should be noted, however, that some flavonoids that carry a single hydroxyl group in their B ring can be *o*-hydroxylated by human cytochrome P450 (CYP) enzymes to form catechols within cells. For instance, CYP1 has been shown to catalyze the hydroxylation of kaempferol in B-3′, converting it into quercetin, and that of galangin in B-4ʹ, converting it into kaempferol [85,176,177]. Another example is the demethylation of 4′-methoxyflavone catalyzed by human CYP1B1.1 and CYP1B1.3, which initially leads to the formation of 4′-hydroxyflavone and subsequently to that of 3′,4′-dihydroxyflavone [178]. Thus, it appears that, in humans, the oxidation of flavonoids can take place via reactions catalyzed by CYP enzymes. These enzymes, however, rather than inducing the oxidative consumption of the redox-active phenolic of the flavonoids, are able to catalyze the incorporation of one or more hydroxyl groups in benzene rings of the flavonoid structure [177]. Although a greater number of hydroxyl groups in the structure of phenolics is generally associated with a greater ROS-scavenging potency [179], the extent to which the CYP-hydroxylation of certain flavonoids contribute to enhance the cell’s antioxidant capacity remains to be established.

As described above, when it comes to the ROS-scavenging properties of flavonoids, the oxidation of certain flavonoid structures (i.e., flavonols) is associated with the formation of mixtures of metabolites whose antioxidant activities are largely retained. In view of the ubiquitous distribution and abundance of the flavonol quercetin in edible plants [32,33], and its relatively low toxicity in humans [180], particular attention has been paid to the study of the consequences that the oxidation of this flavonoid brings on its antioxidant properties.

## 5. Oxidation of Quercetin and Its Consequences on Its Antioxidant Properties

**Figure 2:**
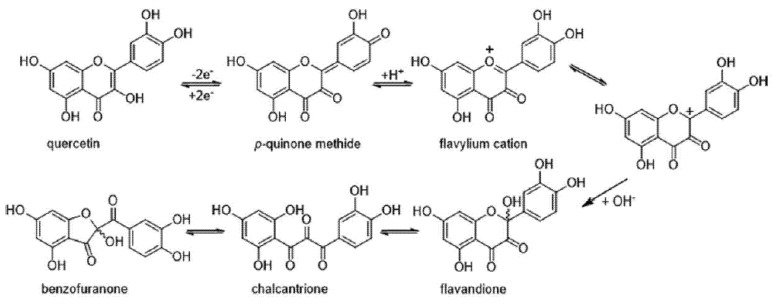
Sequence of chemical structures and reactions proposed to be involved in the oxidative conversion of quercetin into Q-BZF (Reproduced with permission from [57], Copyright © 2017 American Chemical Society).

Among dietary flavonoids, quercetin (5,7,3′,4′-tetrahydroxyflavonol or 3,5,7,3′,4′-pentahydroxyflavone, included in Figure 2) remains one of the most studied molecules [181]. Its early and well-established in vitro capacity to lower ROS formation by scavenging these species [61,182], by chelating redox-active ROS-forming metals [183,184,185], and/or by inhibiting the activity of ROS-generating enzymes such as xanthine oxidase, lipoxygenases, mono-aminooxidase and cyclooxygenase [186,187,188,189,190], has continuously prompted many scientists to engage in the study of its potential as an antioxidant. Regarding its ROS-scavenging property, quercetin possesses key structural features: ortho-dihydroxy substitution in B-ring (catechol structure), which confers high stability to the flavonoid phenoxyl radical via hydrogen bonding or by expanded electron delocalization; the C2–C3 double bond (in conjugation with the 4-oxo group) which determines the coplanarity of the heteroring and participates in radical stabilization via electron delocalization over all three ring systems; and the presence of the 3-OH and 5-OH groups for maximum radical scavenging capacity [191,192].

Quercetin has been shown to be a flavonoid expressing higher antioxidant activity due to the presence of hydroxyl groups and the twisting angle of the B ring [193]. As seen for other flavonoids, however, studies conducted during the last two decades have revealed that the antioxidant effects of quercetin can also arise from actions exerted via the indirect Nrf2 mechanism. In fact, a number of in vitro and in vivo studies have addressed the capacity of quercetin to upregulate, via the Nrf2–Keap1 pathway, the expression of genes that code for the synthesis of antioxidant enzymes such as HO-1 [194], NQO1 [143], and Ɣ-Glu–Cys ligase [145]. However, a question regarding this Nrf2-mediated antioxidant-amplifying effects of quercetin remains as to whether the Nrf2-activating chemical species is the quercetin molecule itself or one or more of its metabolites generated after its oxidation. In an apparently paradoxical manner, different investigators have demonstrated that the ability of quercetin and that of some other limited number of flavonoids to activate Nrf2 correlates well with their intrinsic potential to generate pro-oxidant metabolites, to undergo redox cycling and/or to generate oxidative stress [54,80,159]. Some of the metabolites formed (e.g., *o*-quinones) during the ROS-mediated (or enzymatically induced) oxidation of quercetin exhibit a significant degree of electrophilicity and/or ability to act as pro-oxidant [195,196]. Thus, it would seem that quercetin has a dual antioxidant potential, acting initially, in its non-oxidized form, as an ROS scavenger, and subsequently, after undergoing oxidation, through some of its pro-oxidant metabolites (up-regulating antioxidant responses) [57].

Although quercetin displays a number of bioactivities that do not necessarily arise from its antioxidant properties [197,198,199,200], most of the currently available evidence still supports the contention that a large part of the health benefits associated with its dietary consumption and/or administration are derived from its overall oxidative stress-controlling capacity [43,201,202]. Regarding the latter capacity, it is conceivable that under in vivo conditions, the indirect antioxidant effects of quercetin, increasingly assumed to be the most relevant ones, concur with its direct ROS-scavenging actions. In the latter case, the oxidation of quercetin affects first its 3′ and 4′ hydroxyl moieties in a reaction that leads to the formation of electrophilic intermediates which are endowed with electrophilic and/or pro-oxidant potential [163,167,195]. Subsequently, such intermediates will undergo other oxidative changes that will ultimately affect the flavonoid’s skeleton.

As shown in Figure 2, the two-electron oxidation of quercetin leads to the formation of a *para*-quinone-methide intermediate that, upon protonation, is converted into a flavylium cation; subsequently, the latter compound swiftly undergoes complete hydration to generate the 2,5,7,3′,4′-pentahydroxy-3,4-flavandione. After a ring−chain tautomeric equilibrium, which leads to the formation of a 2,3,4-chalcan-trione intermediate, a polar metabolite identified as 2-(3,4-dihydroxybenzoyl)-2,4,6-trihydroxy-3(2H)-benzofuranone (Q-BZF) is formed [135,203,204,205] (Figure 2). As for other flavonoids, some of the electrophilic intermediates formed during the oxidation of quercetin were implied in the mutagenicity and cytotoxicity reported for this flavonoid in vitro [195,196,206] and in vivo [207]. However, as critically reviewed by Harwood et al. [180], the actual biological significance of such purported toxic actions is highly debatable and lacks any in vivo evidence.

The oxidation of quercetin has been broadly investigated from a chemical standpoint and comprises studies in which its oxidation has been chemically [208,209,210,211], electrochemically [203,211,212,213] and enzymatically induced [135,209,214]. Comparatively, a very limited number of studies have addressed the implications that quercetin oxidation has on its antioxidant properties. In fact, until very recently, only the works by Ramos et al. [215] and by Gülsen et al. [211] had addressed this issue. Using the 2,2-diphenyl-1-picrylhydrazyl (DPPH) assay, Ramos et al. [215] reported that while some quercetin oxidation products retained the scavenging properties of quercetin, others were slightly more potent. Using the DPPH, a hydrogen peroxide, and hydroxyl free radical scavenging assay, Gülsen et al. [211] reported that all quercetin oxidation products were less active than quercetin. From a structural point of view, the oxidative conversion of quercetin into its Q-BZF does not affect rings A and B of the flavonoid but drastically changes ring C, as its six-atom pyran ring is converted into a five-atom furan ring. Taking into consideration the three Bors’ criteria for optimal activity [191], the free radical scavenging capacity of Q-BZF is expected to be significantly less than that of quercetin by the sole fact that its structure lacks the C2–C3 double bond needed for radical stabilization. Based on the latter, it seems reasonable to assume that an ultimate consequence of the oxidation of quercetin would be the relative loss of its original free radical scavenging potency.

Based on the earlier studies of Atala et al. [53], in which the oxidation of several flavonoids resulted in the formation of mixtures of metabolites that largely retained the ROS-scavenging properties of the unoxidized flavonoids, the assumption that oxidation leads to the loss of such activity needed to be revised. In the case of quercetin, the mixtures of metabolites that resulted from its exposure to either alkaline conditions or to mushroom tyrosinase did not differ in terms of their ROS-scavenging capacity, retaining both mixtures near 100% of the original activity. Although the exact chemical composition of the aforementioned oxidation mixtures was not established [53], early studies by Zhou and Sadik [135] and more recently by Heřmánková et al. [205] demonstrated that when it comes to quercetin, regardless of the methods employed to induce its oxidation (i.e., free radical, enzymatic- or electrochemically mediated), an essentially similar set of metabolites is formed.

Prompted by the unexpected retention of the free radical scavenging activity of the mixture of metabolites that arise from quercetin autoxidation (Qox), Fuentes et al. [57] investigated the potential of Qox to protect Hs68 (from a human skin fibroblast) and Caco-2 (from a human colonic adenocarcinoma) cells against the oxidative damage induced by hydrogen peroxide or by the ROS-generating non-steroidal anti-inflammatory drug (NSAID) indomethacin [216,217,218]. When exposed to either of these agents, the quercetin-free Qox mixture afforded total protection with a 20-fold greater potency than that of quercetin (effective at 10 μM). The composition of Qox, as analyzed by HPLC-DAD-ESI-MS/MS, included eleven major metabolites [57]. Each of these metabolites was isolated and assessed for its antioxidant capacity in indomethacin-exposed Caco-2 cells. Interestingly, out of all metabolites, only one, identified as Q-BZF, was able to account for the protection afforded by Qox. The latter was evidenced not only by testing the antioxidant activity of Q-BZF, chromatographically isolated from Qox, but also, after comparing the activity of Qox with that of a Qox preparation from which Q-BZF was experimentally removed by chemical subtraction. Remarkably, the antioxidant protection afforded by the isolated Q-BZF was seen at a 50 nM concentration, namely at a concentration 200-fold lower than that of quercetin [57].

To the best of our knowledge, there are no reports in the literature of any flavonoid or flavonoid-derived molecule capable of acting as antioxidant within cells at such extremely low concentrations. The possibility that such a difference in intracellular antioxidant potency being explained in terms of a 200-fold difference in ROS-scavenging capacity is extremely low since; in addition to lacking the double bond present in ring C of quercetin, Q-BZF does not differ from quercetin in terms of the number and position of their phenolic hydroxyl groups. Considering the extremely low concentration of Q-BZF needed to afford protection against the oxidative and lytic damage induced by hydrogen peroxide or by indomethacin to Hs68 and Caco-2 cells, Fuentes et al. [57] proposed that such effects of Q-BZF could be exerted via Nrf2 activation. Regarding the potential of the Q-BZF molecule to activate Nrf2, several chalcones have already been shown to act as potent Nrf2 activators [219,220]. The electrophilic carbonyl groups of chalcones, including those in the 2,3,4-chalcan-trione intermediate of Q-BZF formation (Figure 2), could be able to oxidatively interact with the cysteinyl residues present in Keap1, the regulatory sensor of Nrf2. Interestingly, an upregulation of this pathway has already been established for quercetin [143,144,145]. Considering the fact that the concentration of Q-BZF needed to afford antioxidant protection is at least 200-fold lower than that of quercetin, and that Q-BZF can be generated during the interaction between quercetin and ROS [135,208], one might speculate that if such a reaction took place within ROS-exposed cells, only one out of 200 hundred molecules of quercetin would be needed to be converted into Q-BZF to account for the protection afforded by this flavonoid—though the occurrence of the latter reaction in mammalian cells remains to be established.

**Figure 3:**
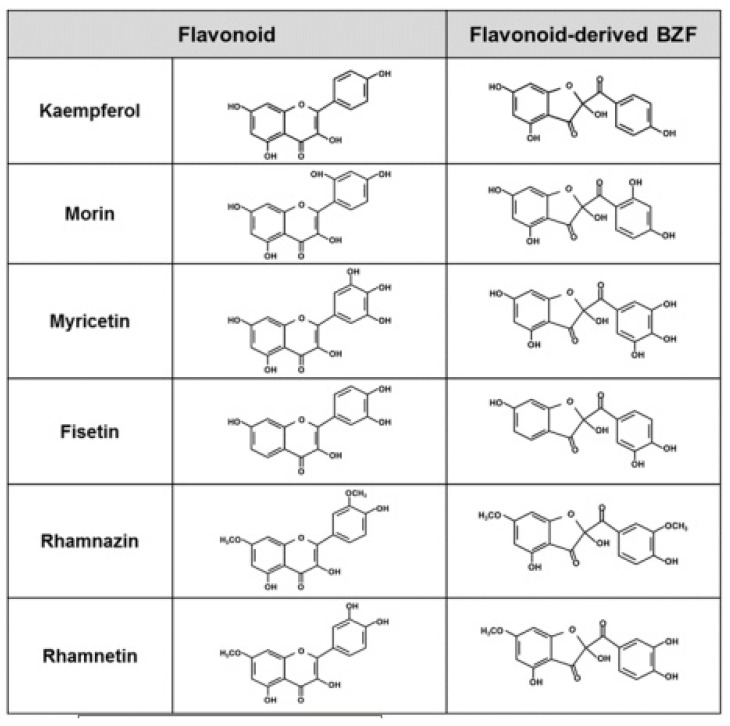
Chemical structures of flavonoids and their corresponding 2-(benzoyl)-2-hydroxy-3(2H)-benzofuranone derivatives.

Interestingly, in addition to quercetin, several other structurally related flavonoids have been reported to undergo chemical and/or electrochemical oxidation that leads to the formation of metabolites with structures comparable to that of Q-BZF. Examples of the latter flavonoids are kaempferol [203,221], morin and myricetin [221], fisetin [221,222,223,224], rhamnazin [225] and rhamnetin [226] (Figure 3). The formation of the 2-(benzoyl)-2-hydroxy-3(2H)-benzofuranone derivatives (BZF) corresponding to each of the six previously mentioned flavonoids requires that a quinone methide intermediate be formed, follows a pathway comparable to that of the Q-BZF (Figure 2), and leads to the formation of a series of BZF where only the C-ring of the parent flavonoid is changed [203,225]. From a structural requirement perspective, the formation of such BZF is limited to flavonols and appears to require, in addition to a hydroxy substituent in C3, a double bond in the C2–C3 and a carbonyl group in C4 (i.e., the basic features of any flavonol), the flavonol possesses at least one hydroxyl group in their ring B [203,221,223]. Based on the already established large increase in antioxidant potency described for quercetin and Q-BZF, it is possible to hypothesize that an amplification of the antioxidant potency could also be seen with the BZF known to be derived from the chemical oxidation of the six previously mentioned flavonols. Our ongoing preliminary work supports such a hypothesis (data not shown), and suggests the emergence of the BZF as a novel group of antioxidants whose intracellular action is exerted with a superior potency compared to that of their precursors. In the perspective of using the Q-BZF, and eventually other BZF, as an antioxidant, it is particularly interesting that the oxidation of quercetin has already been reported in cells of the outer scales of onions (*Allium cepa* L. cepa group) where, in addition to high concentrations of quercetin (of which 87% occurs as aglycone) [227], the Q-BZF occurs [228].

## 6. Onion Peel as a Natural Source of Q-BZF

Considering the notably high antioxidant potency of Q-BZF, its occurrence in the dry peels of onions [228] and the fact that this metabolite can be easily formed during the exposure of quercetin to polyphenol-oxidase [53,214], Fuentes et al. [229] explored by HPLC-DAD-ESI-MS/MS the occurrence of Q-BZF in the peel and/or flesh of a large number of quercetin-rich plant foods, including almond, apples, capers, chives, clove, curcuma, white garlic, ginger, goji, mushrooms, yellow onions, purple onions, oregano, potatoes, radishes, yellow shallots, purple shallots, spinach and walnuts [32]. In addition to corroborating the early finding of Ly et al. [228], these authors found that, among all the other food plants studied, Q-BZF only occurs in shallots (*Allium cepa* L. aggregatum group) and, as in onions, also limited to its dry outer scales. While the outer scales of onions and shallots may serve to protect the bulb of these foods against pathogens by providing a both physical and biochemical barrier, the actual reason for which Q-BZF is only contained in these two plant foods and its presence is restricted to the outer scales remains to be established.

**Figure 4:**
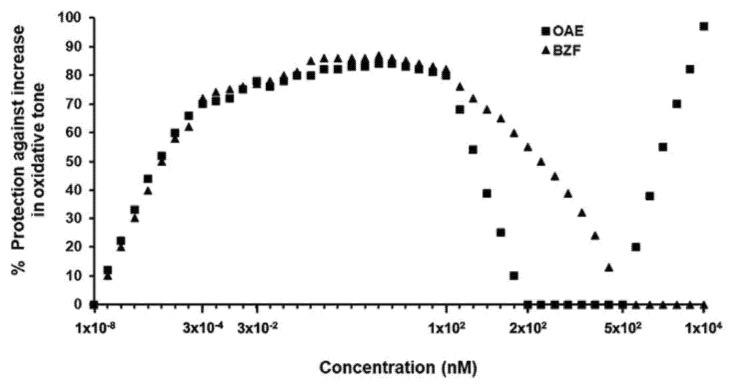
Antioxidant effects of increasing concentrations of Q-BZF present in either a pure Q-BZF preparation (▲) or an onion aqueous extract (OAE) (■) (Reproduced with permission from [229], copyright 2020 Elsevier).

The dry peels of onions, generally discarded as a waste of onion consumption and processing, represents in Europe part of the 450,000 tons of onion solid waste produced yearly [230,231]. Taking advantage of the natural presence of Q-BZF in the outer scales of onions and the fact that this compound has emerged as a particularly potent antioxidant, Fuentes et al. [229] recently developed an aqueous extract from such plant material (OAE). Standardized in terms of its Q-BZF, quercetin and other phenolic contents, OAE was demonstrated to protect Caco-2 cells against oxidative stress (i.e., 2′,7′-dichlorodihydrofluorescein oxidation), and the mitochondrial (i.e., tetrazolium salt reduction-inhibition) and lytic (i.e., lactate dehydrogenase leakage) damage induced by indomethacin, a nonsteroidal anti-inflammatory drug (NSAID). Notably, an antioxidant protection of 65% was seen at a concentration of Q-BZF in OAE as low as 0.03 nM, with a maximum protection of near 85% within the 10–100 nM concentration range (Figure 4).

As shown in the figure, the antioxidant effects of OAE are described by a concentration-dependent curve that was fully overlapped by another curve that described the protection afforded by a pure Q-BZF preparation. According to the same authors [229], such protection was totally lost after the selective chemical subtraction of Q-BZF from OAE, revealing that the ability of the extract to protect cells resides in the presence of Q-BZF in its composition and, that within the aforementioned range of Q-BZF concentrations, any component other than Q-BZF would not contribute to its antioxidant effectiveness. Interestingly, beyond the 100 nM Q-BZF concentration, the protection afforded by the extract and by pure Q-BZF started to swiftly decline, to reach zero at a Q-BZF concentration of 200 nM in OAE and at a 500 nM concentration for Q-BZF. The biphasic concentration-dependent behavior of the antioxidant protection suggests that Q-BZF triggers a “para-hormetic” [42] or hormetic [232] response, where this molecule is able to induce opposite biological effects at different concentrations [233]. Presumably, the oxidized metabolite of quercetin efficiently increases the antioxidant cell capacity at low concentrations and promotes such an effect less efficiently, to reach zero at higher concentrations.

More recently, the ability of Q-BZF, as a pure compound or as part of OAE, to protect Caco-2 cells against the oxidative stress and lytic damage induced by indomethacin was extended to several other NSAIDs [234]. Assessing the protective potential of Q-BZF and/or OAE against the latter agents responds to the lagging need to effectively prevent or ameliorate the adverse gastrointestinal side effects associated with their administration. Such effects comprise a damage that typically begins in the gastric mucosa and that subsequently generates ulcers, hemorrhages and perforations [235]. However, various studies conducted in humans have demonstrated that the duodenal and colonic mucosa are also affected and in an almost similar proportion [236,237]. Although the precise pathogenic mechanism(s) by which NSAIDs induce damage to the gastric and small intestinal mucosa has not been fully established [238], at the cellular level, the co-occurrence of mitochondrial dysfunction and oxidative stress has emerged as a key, early and common molecular event [239,240,241]. Particular attention has been paid to the functional consequences associated with the oxidative stress that affects cells from intestinal epithelia, as the latter leads to alterations of their intercellular tight junctions [242,243] and subsequently, to the loss of the intestinal barrier function [242,244].

The transepithelial electrical resistance (TEER) of monolayers of Caco-2 cells (a human colon epithelial cancer cell line) is a parameter widely used to anticipate the changes in the intestinal barrier function that would take place in vivo [245]. When these cells are grown on a semipermeable filter, they spontaneously differentiate to form a confluent monolayer that structurally and functionally resembles the small intestinal epithelium. As recently demonstrated by Fuentes et al. [234], the simultaneous addition of OAE (containing 100 nM of Q-BZF) to Caco-2 cell monolayers exposed to indomethacin, diclofenac, piroxicam, metamizole or ibuprofen, each added at a concentration that elicited an identical degree of oxidative stress, effectively prevented (by 84–86%) the oxidative stress induced by these agents. However, relative to its antioxidant efficacy, the protection afforded by OAE against the loss of TEER induced by these NSAIDs was highly dissimilar, ranging from 18% (against piroxicam) to 73% (against indomethacin). Fuentes et al. [234] reported that, when correlating both protections, an R^2^ value of 0.087 was obtained, suggesting that the ability of Q-BZF to prevent the oxidative stress is not mechanistically related to its—uneven and only limited—ability to protect the monolayers against the loss of barrier function induced by the former agents. Furthermore, Fuentes et al. [234] observed that, in addition to inducing oxidative stress, the five NSAIDs were able to induce, though to a different extent, the activation of the pro-oxidant and pro-inflammatory nuclear expression factor, nuclear factor kappa B (NF-κB) in monolayers of Caco-2 cells. Interestingly, while OAE fully prevented the NF-κB activation induced by indomethacin, it exerted no inhibitory effect on that induced by the four other NSAIDs, suggesting that the inhibition of NF-κB activation is not necessary to prevent the increase in TEER induced by the latter agents. Although the activation of NF-κB can be both a cause and a consequence of the genesis of ROS [246], in the case of indomethacin, Mazumder et al. [247] recently reported that this NSAID activates the atypical zeta isoform of protein kinase C (PKCζ), which phosphorylates MAPK p38 [248], which in turn activates NF-κB [249]. This nuclear factor can also be activated by different PKC, and this activation can be mediated by ROS [250]. Since indomethacin-induced NF-κB activation may be directly attributed to an increase in ROS or to an indirectly promoted PKCζ activation by the same species, the inhibition of NF-κB activation by Q-BZF could either be attributed to a direct activation-inhibiting action on PKCζ or to an indirect ROS-removing action via Nrf2 activation.

In line with the in vitro protection exerted by Q-BZF or by OAE against the increased paracellular permeability of Caco-2 monolayers induced by indomethacin [234], the capacity of OAE to protect in vivo against the loss of intestinal barrier function induced by the same agent was recently described in rats [251]. In their studies, Fuentes et al. [251], assessing the intestinal permeability using the non-digestible probe 3-5-kDa dextran conjugated with fluorescein isothiocyanate (FITC dextran), observed that the oral administration of Q-BZF (80 μg/Kg body weight) as OAE completely abolished the 30-fold increase in the concentration of FITC dextran seen in the serum of rats simultaneously given indomethacin (40 mg/Kg body weight). This effect was found to be dose-dependent and largely conserved (by 85%) when OAE was given 180 min prior to indomethacin. As previously observed by the same authors in vitro [234], the in vivo observed intestinal barrier function-protective effect of OAE was accompanied by a full prevention of the NF-κB activation and of the increase in the inflammatory parameters interleukine-8 and myeloperoxidase that are typically elevated in the duodenal mucosa of animals given indomethacin [252,253]. It is noteworthy that OAE administration did not alter the basal intestinal mucosa NF-κB levels in animals given no indomethacin. Since deregulated NF-κB activation is a significant causal factor in the pathogenesis of multiple chronic inflammatory diseases [254,255], the ability Q-BZF to prevent the activation of NF-κB opens the possibility of considering the exploration of its therapeutic potential in such types of disorders. With regard to the latter contention, it is worth mentioning the fact that vast literature supports the use of quercetin, the precursor of Q-BZF, as a promising therapeutic strategy to manage several inflammation-related chronic diseases [256]. On the other hand, the administration of Q-BZF, as part of OAE, to the indomethacin given rats was associated with a 21-fold increase in Nrf2 in duodenal mucosa, and a 7-fold and 9-fold increase in the activity of the antioxidant enzymes HO-1 and NQO1, respectively. Such results are in line with a number of studies showing that Nrf2 plays a pivotal role in maintaining the integrity of the intestinal barrier function by suppressing the oxidative stress that downregulates the expression of tight junction proteins that are key in the regulation of paracellular permeability [257]. Based on the former findings, Fuentes et al. [251] proposed that the intestinal epithelial barrier function-protective effect of OAE would involve a dual action of Q-BZF, on the one hand inhibiting the activation of NF-κB induced by indomethacin, and on the other hand inducing the activation of Nrf2. Although the mechanism by which Q-BZF activates Nrf2 remains to be elucidated, one might speculate that it may be related to that of its precursor quercetin, whose capacity to activate Nrf2 and protect the intestinal epithelia against ROS has already been well described [258].

At least from a theoretical point of view, it is worth mentioning the recent work by Vásquez-Espinal et al. [259], who used molecular docking calculations. These authors concluded that compared to quercetin, the stability of the interaction of Q-BZF with the Keap1 kelch domain of Nrf2 was more favorable, thus suggesting a superior potential of the oxidized metabolite to act as an inhibitor of the protein–protein interaction between Keap1 and Nrf2. The modulating role that quercetin and other polyphenols play in the maintenance of the intestinal barrier function [260,261,262,263] suggested that the potential of Q-BZF would not be limited to protecting against the loss of such function induced by NSAID but also that it may contribute to the favorable modulation of its maintenance.

## 7. Conclusions

Faced with the question of whether flavonoids lose, conserve or enhance their antioxidant properties after undergoing oxidation, the current evidence reveals that, at least in the case of certain flavonoids, the mixtures of metabolites that result from their oxidation could conserve, though to a different extent, the ROS-scavenging/reducing capacity of their non-oxidized precursors. Furthermore, in the case of some flavonoids whose oxidation leads to their conversion into pro-oxidant and/or electrophilic metabolites (intermediates or final metabolites), there is increasing evidence to support the concept that through the latter species, such flavonoids would be able to act as an antioxidant, indirectly, through Nrf2 activation. An emerging and noteworthy example of the latter is that of quercetin whose oxidation leads to the generation of Q-BZF, a metabolite that was recently found to be two-to-three orders of magnitude more potently antioxidant than its precursor within cells. The latter metabolite naturally occurs in specific tissues of onions and shallots but not in many of the quercetin-rich plant foods studied to date. In vitro studies conducted with Q-BZF as a pure compound and as part of an aqueous extract obtained from the outer scales of onions revealed the capacity of Q-BZF to protect Caco-2 cells against oxidative stress, mitochondrial and lytic damage induced by ROS such as hydrogen peroxide or NSAIDs. The use of NSAIDs as ROS-generating agents has opened the possibility of projecting the potential use of Q-BZF (and OAE) for protecting against some of the more serious adverse gastrointestinal effects associated with the use of NSAIDs. Within such a conceptual frame of particular interest, there has been the demonstration that nanomolar concentrations of Q-BZF (or Q-BZF contained in OAE) protect Caco-2 monolayers against the oxidative stress and the increase in paracellular permeability induced by NSAIDs. Towards the same aim, studies conducted in rats have recently demonstrated that the loss of epithelial barrier function induced by indomethacin is totally abolished by the oral administration of extremely low doses of Q-BZF contained in OAE. Although the exact mechanisms underlying the intestinal barrier function-protecting effect of Q-BZF remains to be elucidated, the above in vivo studies revealed that such protection might be mechanistically associated with the in vivo ability of the Q-BZF-containing extract to upregulate the activity of certain antioxidant enzymes through the Nrf2 pathway and to abolish the indomethacin-induced activation of NF-κB. This dual capacity of Q-BZF warrants further evaluation under diverse conditions in which controlling the oxidative stress and/or preventing the activation of NF-κB appear to be important for the prevention of certain pathologies.

## Figures and Tables

**Table 1:** Phenol-compromising reactions. As exemplified for quercetin (Q), the main reactions that affect the redox-active phenol moieties of quercetin are listed. In addition, the chemical nature of some of the formed metabolites and the impact that the phenol-compromising reactions can have on the antioxidant properties of the metabolites are described.

Phenol Compromising Reactions	Metabolites	Impact on Antioxidant Potency
**O-Glycosylation** (in plants)	Glycosides (e.g., Q-3-O-glucoside; Q-4′-O-glucoside; 3,4′-O-diglucoside; Q-5-O-glucoside and Q-7-O-glucoside)	In general, these metabolites have less ROS-scavenging potency than their corresponding aglycones
**O-Deglycosylation** (in human intestine/colon)	Quercetin O-deglycosylated in C3, C4′, C5 or C7	The ROS-scavenging potency of O-deglycosylated metabolites is, in most cases, considerably higher
**Biotransformation** (in human intestine/liver/kidney)	Glucuronides (e.g., Q-3-O- and Q-7-O-glucuronides) Sulphates (e.g., Q-3-O-and Q-3′-O-sulphates) Methyl ethers (e.g., Q-3-O- and Q-3′-O-methyl)	These metabolites have, in general, less ROS scavenging/reduction potency but in some particular cases are able to up-regulate the endogenous antioxidant capacity
**Metabolic Degradation** (in human microbiota)	Simple phenolics (e.g., 3,4-dihydroxy-benzoic and 3,4-dihydroxyphenylacetic acids) Deglycosylated flavonoids (e.g., quercetin aglycone)	In general, these metabolites maintain the original ROS-scavenging potency
**Oxidative Consumption** (in plants/possibly in human)	Q-BZF as a mayor oxidation-derived metabolite 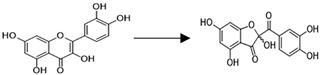	Q-BZF, and possibly other flavonol-derived BZF, maintain their ROS-scavenging potency and show a markedly higher capacity to upregulate the Nrf2-mediated endogenous antioxidant capacity

## Abbreviations

ARE	antioxidant response elements
BZF	2-(benzoyl)-2-hydroxy-3(2H)-benzofuranone derivative(s)
Caco-2	human colonic adenocarcinoma
CAT	catalase
CYP	cytochrome P450
DPPH	2,2-diphenyl-1-picrylhydrazyl
EpRE	electrophile response elements
FITC dextran	3–5-kDa dextran conjugated with fluorescein isothiocyanate
Ɣ-Glu–Cys ligase	gamma glutamate–cysteine ligase
GI	gastrointestinal
GSH	reduced glutathione
GSHpx	glutathione peroxidase
GSSGred	glutathione reductase
HO-1	heme oxygenase-1
Keap1	Kelch-like ECH-associated protein 1
NF-κB	nuclear factor kappa B
NQO1	NAD(P)H:quinone oxidoreductase 1
Nrf2-Keap1	nuclear factor (erythroid-derived 2)-like 2
NSAID	non-steroidal anti-inflammatory drugs
OAE	onion peel aqueous extract
PKC	protein kinase C
PKCζ	protein kinase C zeta type
Q-BZF	2-(3,4-dihydroxybenzoyl)-2,4,6-trihydroxy-3(2H)-benzofuranone
Qox	quercetin oxidation mixture
ROS	reactive oxygen species
SOD	superoxide dismutase
TEER	transepithelial electrical resistance
